# Incidence and Risk Factors for Extensively Drug-Resistant Tuberculosis in Delhi Region

**DOI:** 10.1371/journal.pone.0055299

**Published:** 2013-02-04

**Authors:** Chhavi Porwal, Amit Kaushik, Nayani Makkar, Jayant N. Banavaliker, Mahmud Hanif, Rupak Singla, Anuj K. Bhatnagar, Digambar Behera, Jitendra Nath Pande, Urvashi B. Singh

**Affiliations:** 1 Department of Microbiology, All India Institute of Medical Sciences, New Delhi, India; 2 Rajan Babu Institute for Pulmonary Medicine and Tuberculosis, Delhi, India; 3 New Delhi TB Centre, New Delhi, India; 4 Lala Ram Sarup Institute of Tuberculosis and Respiratory Disease, New Delhi, India; 5 Sitaram Bhartia Institute of Science and Research, New Delhi, India; St. Petersburg Pasteur Institute, Russian Federation

## Abstract

**Background:**

India with a major burden of multidrug-resistant tuberculosis (MDR-TB) does not have national level data on this hazardous disease. Since 2006, emergence of extensively drug-resistant TB (XDR-TB) is considered a serious threat to global TB control. This study highlights the demographic and clinical risk factors associated with XDR-TB in Delhi.

**Methods:**

The study was conducted during April 2007 to May 2010. Six hundred eleven MDR-TB suspects were enrolled from four tertiary care hospitals, treating TB patients in Delhi and the demographic details recorded. Sputum samples were cultured using rapid, automated liquid culture system (MGIT 960). Drug susceptibility testing (DST) for Rifampicin (RIF) and Isoniazid (INH) was performed for all positive M. tuberculosis (M.tb) cultures. All MDR-TB isolates were tested for sensitivity to second-line drugs [Amikacin (AMK), Capreomycin (CAP), Ofloxacin (OFX), Ethionamide (ETA)].

**Results/Findings:**

Of 611, 483 patients were infected with MDR *M. tuberculosis* (*M.tb*) strains. Eighteen MDR-TB isolates (3.7%) were XDR *M.tb* strains. Family history of TB (p 0.045), socioeconomic status (p 0.013), concomitant illness (p 0.001) and previous intake of 2^nd^ line injectable drugs (p 0.001) were significantly associated with occurrence of XDR-TB. Only two of the patients enrolled were HIV seropositive, but had a negative culture for *M. tuberculosis*. 56/483 isolates were pre-XDR *M. tuberculosis*, though the occurrence of pre-XDR-TB did not show any significant demographical associations.

**Conclusions:**

The actual incidence and prevalence rate of XDR-TB in India is not available, although some scattered data is available. This study raises a concern about existence of XDR-TB in India, though small, signaling a need to strengthen the TB control program for early diagnosis of both tuberculosis and drug resistance in order to break the chains of transmission.

## Introduction

India has the maximum burden of tuberculosis (TB) in the world. World Health Organization (WHO) estimated 8.7 million incident cases and 12 million prevalent cases worldwide in 2011. India and China accounted for almost 40% of the world’s TB cases. Globally, there were an estimated 630 000 cases of MDR-TB (range, 460 000–790 000) among the world’s 12 million prevalent cases of TB in 2011. Worldwide, 3.7% of new cases and 20% of previously treated cases were estimated to have MDR-TB. India, China, the Russian Federation and South Africa have almost 60% of the world’s cases of MDR-TB [1].

Globally, emergence of drug resistance is a dangerous alarm. The increase in the incidence of MDR-TB and the emergence of XDR-TB presents tremendous challenges to the global efforts to battle tuberculosis. MDR-TB, defined as resistance to both INH and RIF, is difficult to cure and requires prolonged treatment with expensive and often toxic multidrug regimens. XDR-TB is defined as resistance to at least INH and RIF (MDR-TB) with additional resistance to any fluoroquinolone and at least one of three injectable anti-TB drugs (AMK, CAP or Kanamycin (KAN) [2]–[3], and is known to emerge from MDR-TB, with the acquisition of further drug resistance mutations. Recently published studies and a systematic review have shown that XDR-TB is associated with higher probability of failure and death, and lower probability of treatment success than MDR-TB [2].

Rapid methods enabling accurate susceptibility testing of first-line and second-line drugs are critical for the early diagnosis of MDR-TB and XDR-TB and the initiation of effective regimens [4]–[5]. We report the detection of XDR-TB from statistically significant number of MDR suspects (by 5% level of significance) from Delhi. We screened 611 MDR suspects from 2007 to 2010 and identified isolates meeting the criteria for MDR and XDR-TB [6]. This study points to the existence of XDR-TB in Delhi, India and highlights the lacunae of delayed diagnosis of drug resistance under the program conditions as many of the XDR-TB patients would have initially had MDR-TB that slowly progressed to XDR-TB.

## Materials and Methods

### Sample Size Calculation

Some previous reports from India have shown that close to 5% of the MDR-TB strains are XDR-TB [7]–[12]. Assuming that the prevalence of XDR-TB patients among MDR-TB patients is 5% and it varies from 2%–8%. Considering 5% alpha error (ά) and permissible error (d) as 2%, we had to enroll at least 475 MDR-TB patients. The sample size was calculated by using the standard formula.



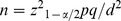



Where Z^2^_1−α/2_ is the standard normal deviate at α level of significance (1.96), p is the prevalence of particular group; q = 1−p; d is permissible error at 5% level of significance.

### Ethics Statement

This study was approved by the institutional ethics review committee, (IEC approval no. T-10/31.10.2008) AIIMS, New Delhi. Written informed consent was obtained from each patient prior to collection of sputum sample.

### Collection of Clinical Isolates of *M. tuberculosis*

A total of 611 patients were enrolled and sputum samples collected from four different tertiary care hospitals treating TB in Delhi. All patients were enrolled on the basis of inclusion criteria (Cases clinically suspected to be suffering from drug resistant tuberculosis/category I or category II failure cases). Patient information was collected in a standard proforma. Demographic data was collected viz. gender, age, address, employment, economic status, literacy, living conditions, household contacts, professional work contacts, chest radiological findings, any other associated illness and HIV infection.

### *M. tuberculosis* Culture and Drug Susceptibility Testing by MGIT 960

All samples were processed using NALC-NaOH method [13] and smears were examined after Ziehl–Neelsen staining. Processed samples were inoculated into MGIT 960 non-radiometric automated isolation system (Becton Dickinson, Sparks, MD, USA). MGIT tube was supplemented with 0.8 ml of Oleic Acid-Albumin-Dextrose-Catalase (OADC) along with mixture of five antibiotics; Polymixin B, Amphotericin B, Nalidixic acid, Trimethoprim, and Azlocillin (PANTA) and 0.5 ml of decontaminated sample. *M. tuberculosis* complex and non-tubercular mycobacteria (NTM) were differentiated using p-nitrobenzoic acid (PNB) test (as per the manufacturer’s instructions) [14].

The standard protocol for DST of RIF and INH in the MGIT 960 was followed according to the manufacturer’s instructions [14]. Briefly, to each 7 ml MGIT tube, 0.8 ml of supplement (MGIT 960 SIRE supplement) and 0.1 ml of the drug stock (Becton Dickinson, USA) (final concentrations were 0.1 µg/ml for INH and 1 µg/ml for RIF) solution were aseptically added and finally 0.5 ml of the test inoculum was added. For each isolate, a growth control (GC) with growth supplement but without drug was included. To prepare GC the inoculum was prepared by pipetting 0.1 ml of the test inoculum with 10 ml of sterile saline to make a 1∶100 dilution; 0.5 ml of GC inoculum was added to a drug free MGIT tube. All of the inoculated tubes were placed into MGIT 960 instrument on the same day.

DST for second line drugs was performed on MDR-TB isolates by using 5 µg/ml of ETA, 2.5 µg/ml of CAP, 2 µg/ml of OFX and 1.0 µg/ml of AMK(Sigma-Aldrich, St.Louis, Mo, USA) [15]–[17] in MGIT 960. XDR-TB isolates were further tested by 2.5 µg/ml of KAN and 2.0 µg/ml of Levofloxacin (LVF) (Sigma-Aldrich, St. Louis, Mo, USA) [15], [17]. All the tubes were tightly recapped and mixed well. All inoculated drug-containing and GC tubes were placed in the DST set carrier and entered into the MGIT 960 instrument as ‘unknown drugs’ using the AST (antimicrobial susceptibility testing) entry feature.

### Case Definition

MDR-TB was defined as tuberculosis disease caused by a strain of *M. tuberculosis* that was resistant to at least RIF and INH. Pre-XDR was defined as disease caused by a strain resistant to RIF and INH and either a fluoroquinolone or a second-line injectable drug, but not both [18]. XDR-TB was defined as TB with resistance to at least RIF, INH, a fluoroquinolone and one of three second-line injectable drugs (CAP, AMK Or KAN) [19].

### Analysis

The following risk factors were analyzed: gender, age, smoking, alcohol use, socioeconomic status, residence location, co-morbidities, previous treatment history, BCG status, family TB history, previous use of fluoroquinolone or injectable drugs and cavitation on chest X-ray. Data was analyzed using the SPSS software, version 10.0 (SPSS Inc, Chicago, IL, USA). Comparisons of categorical variables were performed using the Pearson Chi-square test to compare different groups. Non-categorical variables (age and socioeconomic status) were analyzed by Kruskal-Wallis test. A p-value of <0.05 was considered as statistically significant.

## Results

### Characteristics of the Study Population

**Figure 1:**
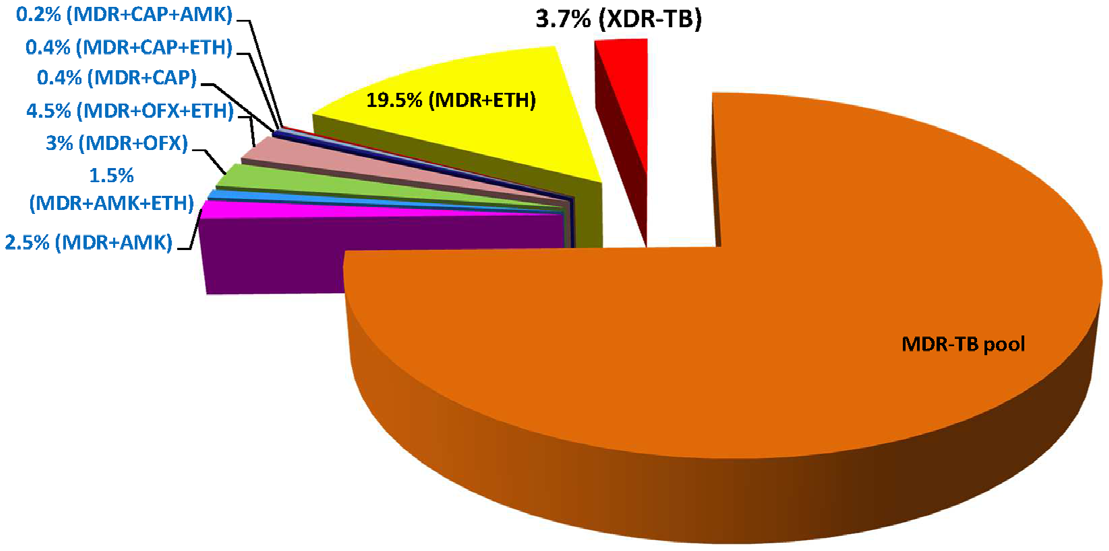
Diagram shows the distribution of multidrug-resistant/pre-extensively drug-resistant and extensively drug-resistant *M. tuberculosis* isolates with additional resistance profile.

Six hundred eleven patients with clinical suspicion of drug resistant TB (Cat I/II treatment failure cases) were enrolled. DST for RIF and INH on these patients showed 483 to be infected with MDR-TB strains. Of 483 MDR-TB isolates, 18 (3.7%) strains were found to be XDR-TB, 7.5% MDR-TB strains were resistant to OFX alone and 5% MDR-TB strains were resistant to second line injectable drugs (AMK and CAP), termed as pre-XDR (Fig. 1).

All XDR-TB (n = 18) isolates were tested for sensitivity to KAN and LVF and had the same resistance pattern as with AMK and OFX (Table 1), with one exception, an isolate sensitive to AMK was found resistant to KAN.

**Table 1:** Drug resistance pattern of XDR-TB patients.

Strain ID	Drug susceptibility testing (DST)				
	Aminoglycoside		Fluroquinolone		Polypeptide
	Amikacin	Kanamycin	Ofloxacin	Levofloxacin	Capreomycin
M 1	R	R	R	R	S
M2	R	R	R	R	R
M 3	R	R	R	R	S
M 4	R	R	R	R	R
M 5	S	S	R	R	R
M 6	R	R	R	R	S
M 7	R	R	R	R	R
M 8	S	S	R	R	R
M 9	S	S	R	R	R
M 10	S	S	R	R	R
M 11	R	R	R	R	R
M 12	R	R	R	R	R
M 13	R	R	R	R	S
M 14	S	R	R	R	R
M 15	R	R	R	R	S
M 16	R	R	R	R	S
M 17	S	S	R	R	R
M 18	R	R	R	R	S

Note: R = Resistant, S = Sensitive.

Of 611 enrolled patients, 2 were HIV sero-positive but were culture negative for *M.tb* and hence were excluded from the study.

Among MDR-TB patients, 36.8% patients were females with the mean age of 31 years (SD 11.2). Fifty percent of MDR patients showed cavitation on Chest X-ray (CXR) and 62% patients had previously taken either 2^nd^ line injectables or fluoroquinolones (Tables 2, 3 and 4).

**Table 2:** Comparison of clinical characteristics (categorical values) of MDR, p-XDR and XDR-TB isolates.

Characteristics		MDR-TB total number (%)	p-XDR-TB total number (%)	XDR-TB total number (%)	p value
Sex	Male	260 (63.1)	25 (47.2)	12 (66.6)	0.072
	female	152 (36.9)	28 (52.8)	6 (33.3)	0.072
Previous TB treatment (n = 483)	Yes	380 (92.2)	51 (96.2)	17 (94.4)	0.551
	No	32 (7.8)	2 (3.8)	1 (5.5)	0.551
Family TB history (n = 483)	Yes	72 (17.5)	11 (20.7)	7 (38.9)	**0.045**
	No	340 (82.5)	42 (79.2)	11 (61.1)	**0.045**
Smoker (n = 483)	Yes	168 (40.8)	20 (37.7)	9 (50.0)	0.658
	No	244 (59.2)	33 (62.2)	9 (50.0)	0.658
Alcohol (n = 483)	Yes	125 (30.3)	14 (26.4)	7 (38.9)	0.604
	No	287 (69.7)	39 (73.6)	11 (61.)	0.604
Residence (n = 483)	Urban	194 (47.0)	23 (43.0)	5 (27.7)	0.612
	Rural	218 (52.0)	30 (56.0)	13 (72.0)	
Co-morbidity (n = 254)	Yes	19 (4.61)	4 (7.5)	6 (33.3)	**0.001**
	No	393 (95.4)	49 (92.4)	12(66.6)	**0.001**
CXR cavitation (n = 254)	Yes	107 (51.7 )	17 (56.6)	10 (58.8)	0.767
	No	100 (48.3)	13 (43.3)	7 (41.9)	0.767
BCG status (n = 163)	Yes	59 (47.5)	12 (52.2)	5 (31.2)	0.397
	No	65 (52.4)	11 (47.8)	11 (68.7)	0.397
Category status					
CATI failed		154 (37.4)	21 (39.6)	5 (27.8)	0.74
CATII failed		209 (50.7)	28 (52.8)	4 (22.9)	0.66
CATIV failed		18 (4.4)	1 (1.8)	7 (38.9)	**0.049**
Not known		31 (7.5)	3 (5.7)	2 (11.1)	
Previously taken FQs(n = 93/163)	Yes	68 (54.8)	15 (65.2)	10 (62.5)	0.586
	No	56 (45.8)	8 (34.7)	6 (37.5)	0.586
Previously taken 2^nd^ line injectable (n = 22/163)	Yes	9 (7.2)	3 (13.0)	10 (62.5)	**0.001**
	No	115 (92.7)	20 (86.9)	6 (37.5)	**0.001**

P values were calculated using chi-square test, values <0.05 considerd as significant.

CXR = Chest X-Ray, MDR-TB = Multi Drug Resistant Tuberculosis, p-XDR-TB = Pre Extensively Drug Resistant Tuberculosis, XDR-TB = Extensively Drug Resistant Tuberculosis, FQS = Fluroquinolones, BCG =  Bacillus Calmette–Guérin.

**Boldface** indicates statistically significant differences.

**Table 3:** P value comparison of patients with history of previous intake of 2^nd^ line injectable drugs among MDR, p-XDR and XDR-TB isolates.

Patients with history of previous intake of 2^nd^ line injectables (n = 22/163)	
Groups	P value
MDR vs. p-XDR	0.352
MDR vs. XDR	**0.000**
p-XDR vs. XDR	**0.001**

P values were calculated using Fisher's exact test, values <0.05 considerd as significant.

MDR-TB = Multi Drug Resistant Tuberculosis, p-XDR-TB = Pre Extensively Drug Resistant Tuberculosis, XDR-TB = Extensively Drug Resistant Tuberculosis.

**Boldface** indicates statistically significant differences.

**Table 4:** Comparison of clinical characteristics (non-categorical values) of MDR, p-XDR and XDR-TB isolates.

	Mean±Standard Deviation			
Characteristics	MDR-TB	p-XDR-TB	XDR-TB	P value
Age	31.7**±**11.27	32**±**12.81	34.4**±**8.9	0.469
Previous TB history (years)	0.97**±**0.15	0.98**±**0.13	0.94**±**0.23	0.677
INR	5840.5**±**8494.5	5020.7**±**6179.2	8000**±**9639.0	**0.013**

P values were calculated using Kruskal-Wallis test, values<0.05 considerd as significant.

MDR-TB = Multi Drug Resistant Tuberculosis, p-XDR-TB = Pre Extensively Drug Resistant Tuberculosis, XDR-TB = Extensively Drug Resistant Tuberculosis, INR = Indian National Rupees.

**Boldface** indicates statistically significant differences.

Among pre-XDR-TB patients (n = 53), 52.8% were females with the mean age of 32 years (SD 12). Fifty six percent of these patients showed cavitation on CXR and 78% patients had a history of taking either fluoroquinolones or one of the 2^nd^ line injectable drugs. Twenty percent patients gave a family history of TB (Tables 2, 3 and 4).

In XDR-TB cases, 33.3% patients were females and the mean age was 34 years (SD 8.9). Seven patients had co-morbidities (*i.e.* diabetes mellitus, myasthenia gravis and thalassemia intermedia) and 38.8% patients gave a history of tuberculosis in their families. Fifty eight percent patients had cavitation on CXR and 81% patients had previously taken either fluoroquinolones or 2^nd^ line injectables (Tables 2, 3, 4 and 5).

**Table 5:** Treatment history of XDR *M. tuberculosis* isolates.

Strain ID	Previous treatment for TB/Previous pattern of drug resistance	*Name of Drugs	**Treatment after DST report	Co-morbidities	Family history/Death	Outcome
M 1	Yes/MDR-TB	KAN, ETA, D-CS,PZA, AX	CAP, CFZ, CLARI, MOX, PAS,High Dose INH	Diabetes mellitus	NH	NA
M2	Yes/MDR-TB	D-CS, ETA, PAS, LVF	CFZ, CLARI, MOX, PAS, Amoxiclav, Linezolid	None	Mother/No	Defaulter
M 3	Yes/TB, DST (2^nd^ line) ongoing	HRZE	CAP, CFZ, CLARI, MOX, PAS, Linezolid, High dose INH	None	NH	Expired
M 4	Yes/MDR-TB	ETH, ETA, D-CS,OFX	CFZ, CLARI, MOX, PAS, Amoxiclav, Linezolid,High dose INH	Myasthenia gravis	Father & brother/Yes	Expired
M 5	Yes/TB, DST (2^nd^ line) ongoing	CAT II DOTS	CLARI, MOX, PAS, Amoxiclav, Linezolid, High dose INH	Thalassemia intermedia	NH	Expired
M 6	Yes/MDR-TB	KAN,ETA, D-CS,PZA	CAP, CFZ, CLARI, MOX, PAS, Linezolid	None	NH	NA
M 7	Yes/MDR-TB	KAN,ETA,ETZ,PAS,PZA	CLARI, MOX, PAS, Amoxiclav, Linezolid, High dose INH	None	Husband/No	Defaulter
M 8	Yes/MDR-TB	KAN, PAS, PZA, ETA, LVF, D-CS	CLARI, CFZ, MOX, Amoxiclav, Linezolid, High dose INH	None	Brother/Yes	Defaulter
M 9	Yes/TB, DST (2^nd^ line) ongoing	CAT II DOTS	CLARI, MOX, PAS, Amoxiclav, Linezolid, High dose INH	Diabetes mellitus	Uncle/No	Defaulter
M 10	Yes/TB, DST (2^nd^ line) ongoing	CAT II DOTS	CLARI, MOX, PAS, Amoxiclav, Linezolid, High dose INH	Diabetes mellitus	NH	Expired
M 11	Yes/MDR-TB	ETA, PAS, PZA, D-CS	CFZ, CLARI, MOX, PAS, Amoxiclav, Linezolid,High dose INH	None	NH	Cured
M 12	Yes/TB, DST (2^nd^ line) ongoing	CATII DOTS	CLARI, MOX, PAS, Amoxiclav, Linezolid, High dose INH	Diabetes mellitus	NH	NA
M 13	Yes/MDR-TB	KAN, ETA,D-CS,PZA,AX	CFZ, CLARI, MOX, PAS, Amoxiclav, Linezolid, High dose INH	None	NH	NA
M 14	Yes/TB, DST (2^nd^ line) ongoing	No record	CFZ, CLARI, MOX, PAS,Linezolid, High dose INH	None	NH	NA
M 15	Yes/MDR-TB	CAP, CFZ, ETA, D-CS, PZA, AX	CAP, CFZ, CLARI, MOX, PAS,	None	NH	NA
M 16	Yes/TB, DST (2^nd^ line) ongoing	CAT II DOTS	CLARI, MOX, PAS, Amoxiclav, Linezolid, High dose INH	None	NH	NA
M 17	Yes/TB, DST (2^nd^ line) ongoing	CAT II DOTS	CLARI, MOX, PAS, Amoxiclav, Linezolid, High dose INH	Spinal tuberculosis	Mother/No	NA
M 18	Yes/TB, DST (2^nd^ line) ongoing	HRZE	CLARI, MOX, PAS, Amoxiclav, Linezolid, High dose INH	Transfusion and jaundice	NH	NA

**Note:** All isolates were pulmonary TB patients.

* The patients were on treatment with enlisted drugs at the time of sample collection.

** The patients were on treatment with enlisted drugs after providing DST report.

MDR-TB = Multi Drug Resistant Tuberculosis, KM = Kanamycin, PZA = Pyrazinamide, ETA = Ethionamide, PAS = Para Amino salicylic acid, LVF = Levofloxacin, D-CS = D-cycloserine, AX = Amoxicillin, ETH = Ethambutol, CAP = Capreomycin, CFZ = Clofazimine, CLARI = Clarithromycin, MOX = Moxifloxacin, INH = Isoniazid, NA = not available, NH = No history.

### Risk Factors Associated with Progression of the Disease

**Figure 2:**
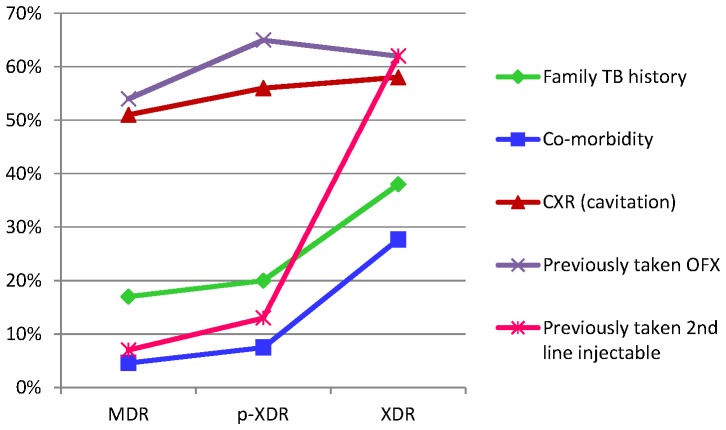
Line diagram shows the correlation of family TB history, co -morbidity, chest X-ray (CXR) findings and patients who have previously taken OFX OR 2^nd^ line injectables among MDR, p-XDR and XDR *M. tuberculosis* isolates. Of these, co-morbidity (p 0.001), family TB history (p 0.045) and previous intake of 2^nd^ line injectable drugs (p 0.001) were significantly associated with progression of the disease. Other two factors (CXR and previously taken OFX) were associated though not significantly.

**Figure 3:**
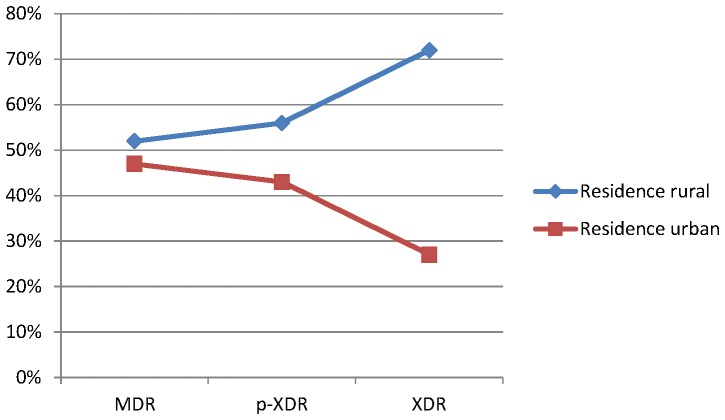
Line diagram shows the correlation of residence (rural and urban) among MDR, p-XDR and XDR *M. tuberculosis* isolates, although the p value was not statistically significant.

Extent of cavitations in chest X-ray seemed to be associated with drug resistance, highest cavitations being associated with XDR-TB (though p value was not statistically significant). Past history of fluoroquinolone intake (though not statistically significant) was also associated with p-XDR/XDR-TB (Tables 2 and 3). Four factors showed significant association with worsening drug resistance, family history of TB (p 0.045), socioeconomic status (p 0.013), co-morbidities (0.001) and previous intake of second line injectable drugs (p 0.001) (Tables 2, 3 and 4). Rural residence seemed to foster XDR-TB while on the contrary there was more MDR-TB and less XDR-TB in urban areas. Figure 2 & 3 shows the precise differentiation of these variables among MDR/p-XDR/XDR-TB isolates.

## Discussion

XDR-TB is a serious global health threat. The emergence of XDR TB reflects a failure to implement the measures recommended in the WHO’s Stop TB strategy. This strategy emphasizes expanding high quality DOTS programme [20].

Emergence of XDR-TB is reported worldwide. Reported prevalence rates of XDR-TB of total MDR cases are: 6.6% overall worldwide, 6.5% in industrialized countries, 13.6% in Russia and Eastern Europe, 1.5% in Asia, 0.6% in Africa and Middle East and 15.4% in Republic of Korea [21]. XDR-TB has been reported from 84 countries by end of 2011; the average proportion of MDR-TB cases with XDR-TB is 9.0% and is significantly associated with worse outcome than MDR-TB [1], [6].

The actual incidence and prevalence rate of XDR-TB in India is not available. A few scattered reports: Mondal *et al* reported 7.4% of MDR strains as XDR [7]; a study from Hinduja Hospital, Mumbai revealed 11% of MDR strains as XDR [11]. Singh *et al* reported 33.3% of MDR TB cases as XDR-TB in a population of HIV sero-positive patients from AIIMS, New Delhi [22]. Sharma *et al* found 2.4% of MDR as XDR-TB cases from Delhi [9]. A study conducted by Paramasivan *et al* reported 4.6% XDR-TB cases in Chennai [8] and another recent study by Khanna *et al* from Delhi reported 5.76% XDR-TB [10]. None of these studies were carried out as per statistical significance considerations of the numbers of MDR TB isolates included.

Development of drug resistance (MDR/p-XDR/XDR) may result due to various reasons: inappropriate treatment regimens (*i.e*. drug selection, duration of treatment and the correct dosage); patient factors (*i.e.* mal-absorption, poor adherence); programme-related factors (*i.e.*, incompetent health personnel, irregular drug supply). In fact, it has been said that the emergence of MDR-TB is evidence of systematic failure of the global community to tackle a curable disease [23].

We designed a cross sectional study and enrolled 611 clinically suspected drug resistant TB patients. Of 611 patients, samples from 483 grew MDR-TB cultures, and eighteen of the 483 MDR-TB isolates were XDR-TB (3.7%). The current study was only designed to study the existence of XDR-TB and the association of risk factors, and not to comment on the prevalence of XDR-TB in India.

Analysis of XDR-TB cases showed that XDR-TB was more frequent among patients with family history of TB (p 0.045) (Table 2). In 33.3% (6/18) of the families of XDR-TB patients, history of TB was recorded, though no information regarding the treatment regimen followed or drug resistance pattern was available. Of these 6 XDR-TB patients, 1 patient expired while 4 were defaulters. The data implies that family contact with TB patients may be one of the main reasons for the spread of the disease. Disease in the family may indicate the role of some genetic or immunological predisposition in families or just the increased transmission due to close and prolonged exposure. Family history of TB could lead to some patients actually getting primary XDR-TB, which may be responsible for florid disease. In addition, compromised care due to social factors such as possible callousness to a challenge that has caused protracted morbidity but no mortality, or loss of faith in the treatment regimens possibly explain the high defaulter rate.

A salient and novel observation in the study was co-morbidities in seven XDR-TB patients. These patients had Diabetes mellitus (n = 4), Myasthenia gravis (n = 1), Thalassemia intermedia (n = 1) and transfusion associated jaundice (n = 1) (Table 5). Patients with an associated pathology showed a higher tendency for acquiring resistance (p 0.001) (Table 2). Patients with co-morbidities often have compromised immunity. Hence we propose a strong role of immune competence in controlling the disease, with or without treatment. A poor immune response would enhance chronicity of disease, and would hence foster accumulation of further mutations and the subsequent selection of a highly resistant clone of bacteria.

It was observed that 83.3% (15/18) of XDR-TB patients were from poor socio-economic status (monthly income, Indian National rupees (INR) <8,000/-) (p 0.013) (Table 4) due to which these patients may not find it affordable to get the necessary tests done, like DST (not part of programmatic services at the time of the study). Other social factors such as poor nutrition, poor standards of living, hence higher exposure to disease, which come with poor socio-economic status may also contribute. Thus we may infer from the study that socio-economic status may be a contributor for developing drug resistant TB.

Interestingly, patients who had taken 2^nd^ line injectable treatment earlier had higher chances of getting XDR-TB (p 0.001) (Tables 2 and 3). Jeon *et al* have reported XDR-TB to be associated with the cumulative duration of previous treatment with second-line TB drugs among subjects in a tertiary care TB hospital in S. Korea [24]. Dalton *et al* have reported that prior use of second-line anti-TB drugs more than quadrupled the risk of extremely drug- resistant tuberculosis (XDR-TB) in an 8-country prospective study [25]. Chan *et al* have demonstrated in a cohort of 174 patients with multidrug- resistant TB that 12 patients with multidrug- resistant TB strains resistant to the fluoroquinolones and streptomycin had significantly better initial and long-term outcomes, compared with 10 patients with extensively drug- resistant TB, hence signaling caution towards misuse of 2^nd^ line injectables [26].

There was a distinct difference in the chances of finding XDR-TB in patients who hailed from a rural *vis a vis* urban background. MDR-TB patients in rural areas had higher chances of disease worsening to lead to XDR-TB; on the contrary, in patients who belonged to urban areas XDR-TB formed a smaller subset of MDR-TB patients. This was an incidental finding and may signal towards ignorance of rural patients towards available treatment options or possibly poor coverage of programmatic services in such areas (Figure 3).

Our data suggests that an inadequate initial drug regimen may be associated with the development of XDR, as we observed, 94.4% XDR-TB patients were previously treated for tuberculosis (Table 2). These patients were already under medication for TB, though their compliance and dosage schedules, sources of medicine could not be ascertained. Disease worsening, accumulation of drug resistance, progression of MDR to XDR could all result from inadequate treatment.

High numbers of p-XDR-TB cases is a cause for great concern. OFX resistance seen in 7.4% of MDR strains and AMK/CAP resistance in 5.9% are only a single mutation away from converting to an XDR-TB strain. A recent study in South Africa raised the concern of those MDR-TB cases that were resistant to a single marker of XDR-TB (either OFX or KAN), as being at great risk of developing XDR-TB if not managed appropriately [27]. Issues about poor/non-response to programmatic management of MDR-TB loom large due to such cases.

The XDR-TB patients were followed up and it was found that 4 (22.2%) expired during treatment, 1(5.5%) was treated successfully, 4 (22.2%) defaulted and 9 (50%) were not traceable. The treatment regimen being given to these patients is given in Table 5.

Our study has a few limitations. Data on CXR, BCG vaccination and intake of injectable/fluoroquinolone were not available for all patients. Patients were not willing to give proper residential information; hence migrants could not be traced for follow up. Patients did not have proper previous medical records; hence it was difficult to explain the poor response or progress to drug resistant TB. The small number of XDR-TB (3.7%) cases was also a limitation. It is likely that a larger patient cohort would have shown a more significant association with various variables, including migrants, co-morbidity, socioeconomic status, age, sex, underlying diseases such as chronic obstructive pulmonary disease or abnormal liver function. Another limitation was that resistance to KAN was not checked in all the isolates and only for XDR-TB isolates. Maus *et al* have cautioned against the faulty practice of generalizing resistance to a class of drugs, e.g., cyclic peptides or aminoglycosides, based solely on the resistance to a single drug in the class [28].

The percentage of MDR and XDR-TB patients that are detected depends on the study design, the sampling frame and the study population. There is a need to reevaluate and recalculate actual prevalence of XDR-TB from different population samples residing in various regions of India. Currently, the rapid diagnosis and treatment of persons with TB, particularly any form of drug- resistant TB, are high priority public health interventions. Effective control of drug resistant TB requires massive scaling-up of culture, DST capability and novel rapid assays to detect drug resistance [29]. DST is recommended universally for new and retreatment TB cases. Early treatment of MDR-TB with drugs reserved only for treating such patients would again contribute significantly to the control; though this would entail strict action to restrict the use of these drugs only for the program. A quick diagnosis of MDR and XDR-TB translates into greater likelihood of patient care and less spread of this potentially lethal strain thus benefiting the individual and the society. In addition, more exhaustive efforts should be made to manage drug resistant TB cases more effectively to improve treatment outcomes of all patients and hence minimize further development of TB resistant to all available drugs.
